# Oligonucleotides conjugated with short chemically defined polyethylene glycol chains are efficient antisense agents

**DOI:** 10.1016/j.bmcl.2014.10.045

**Published:** 2014-12-15

**Authors:** Nasrin Shokrzadeh, Anna-Maria Winkler, Mehrdad Dirin, Johannes Winkler

**Affiliations:** University of Vienna, Department of Pharmaceutical Chemistry, Althanstraße 14, 1090 Vienna, Austria

**Keywords:** Oligonucleotides, Antisense agents, Bioconjugation, Polyethylene glycol

## Abstract

Ligand conjugation is an attractive approach to rationally modify the poor pharmacokinetic behavior and cellular uptake properties of antisense oligonucleotides. Polyethylene glycol (PEG) attachment is a method to increase solubility of oligonucleotides and prevent the rapid elimination, thus increasing tissue distribution. On the other hand, the attachment of long PEG chains negatively influences the pharmacodynamic effect by reducing the hybridization efficiency. We examined the use of short PEG ligands on the in vitro effect of antisense agents. Circular dichroism showed that the tethering of PEG_12_-chains to phosphodiester and phosphorothioate oligonucleotides had no influence on their secondary structure and did not reduce the affinity to the counter strand. In an in vitro tumor model, a luciferase reporter assay indicated unchanged gene silencing activity compared to unmodified compounds, and even slightly superior target down regulation was found after treatment with a phosphorothioate modified conjugate.

## Graphical abstract

**Figure:**
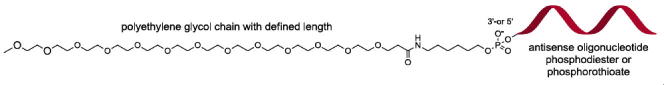

During the last years, the therapeutic development of oligonucleotides has experienced several important advances. For the first time, a systemically applied oligonucleotide, mipomersen, has won market approval.^1^, ^2^ Promising clinical data have been reported for treatment of Duchenne muscular dystrophy by the splice-switching oligonucleotides eteplirsen.^3^ siRNA-based agents have recently entered clinical testing, and many new or optimized oligonucleotide delivery systems have been evaluated preclinically.^4^, ^5^ Thus, good progress has been made in terms of solving the delivery problem for local administration, and also for hepatocyte targets; all clinically successful approaches use systems with preferential accumulation in the liver. In addition, the expanding knowledge of the multifold roles of endogenous small non coding RNAs, above all microRNAs,^6^, ^7^, ^8^, ^9^ have increased the potential therapeutic options and targets of oligonucleotide medicines.

For the delivery of siRNA and miRNA-like oligonucleotides, the limited tolerance of the molecular RNAi machinery all but prevents the use of oligonucleotides that are chemically modified to a degree which allows naked application.^5^ In these cases, encapsulation or complexation with liposomes or nanoparticles or conjugation to uptake-enhancing agents are essentially required.^4^, ^10^ For antisense and splice-switching applications, however, the use of more drastically derivatized compounds, which confer adequate stability against nuclease degradation and favorable binding to plasma proteins, is possible.^1^, ^11^

Recently, we have reported the influence of the attachment of short PEG chains to siRNA oligonucleotide on their gene silencing efficiency.^12^ In contrast to long PEG chains, which result in adequate shielding against nuclease degradation, but reduce the gene silencing effect,^13^ shorter ligands with twelve ethylene glycol units did not diminish the effect. PEGylation even resulted in enhanced gene silencing for an siRNA which had only average efficiency in its wild-type form.^12^

While antisense and siRNA oligonucleotide differ in the molecular machinery, their physicochemical properties are very similar. Antisense oligonucleotides profit from vast possibilities of modifying their chemical structure without significant loss of activity.^11^ Among those modifications, the phosphorothioate backbone has been proven to be particularly useful, because it enhances cell membrane permeation.^14^ Thus, we examined the effect of short PEG chains on the in vitro gene silencing activity both after application with a transfection agent and after unassisted cellular uptake of phosphodiester and phosphorothioate antisense agents.

Aminohexyl linkers were introduced into oligonucleotides targeted at galectin-1 and bcl-2 at the 3′- and 5′-position via a commercially available modified solid phase and a phosphoramidite building block, respectively (Table 1). Oligonucleotide syntheses were carried out using standard DNA synthesis protocols and reagents. For phosphorothioate syntheses, TETD (tetraethylthiuramdisulfide) was used as the sulfurization reagent.^15^ For attachment of a 3′-amino group, amino-on CPG resin was used, and 5′-amino tethers were introduced with a corresponding aminohexyl phosphoramidite. Cleavage and full deprotection were afforded in concentrated ammonia at 55 °C for 16 h. In contrast to the experience with AMA deprotection for siRNA (longer reaction times were necessary for full hydrolysis of the amino protecting group),^12^ the 3′-amino tether was fully deprotected during standard ammonia treatment.

**Table 1:** Oligonucleotide sequences targeted at galectin-1 (**1**–**6**) and bcl-2 (**7**–**10**)

No.	Sequence	Strand	Modification
**1**	TTCGTATCCATCTGGCAGC	AS	—
**2**	*PEG*-TTCGTATCCATCTGGCAGC	AS	5′-PEG
**3**	TTCGTATCCATCTGGCAGC-*PEG*	AS	3′-PEG
**4**	TTCGTATCCATCTGGCAGC	AS	Full PS
**5**	TTCGTATCCATCTGGCAGC-*PEG*	AS	Full PS, 3′-PEG
**6**	GCTGCCAGATGGATACGAA	S	—
**7**	TCTCCCAGCGTGCGCCAT	AS	—
**8**	*PEG*-TCTCCCAGCGTGCGCCAT	AS	5′-PEG
**9**	TCTCCCAGCGTGCGCCAT-*PEG*	AS	3′-PEG
**10**	ATGGCGCACGCTGGGAGA	S	—

These linkers were then used as attachment points for NHS-activated PEG_12_ units (Scheme 1). For a nearly quantitative yield after 2 h reaction time, a 50fold surplus of the NHS-PEG ligand was added from a stock solution in anhydrous DMF to the oligonucleotide dissolved in sodium borate buffer. The unreacted PEG ligand was removed from the mixture by gel filtration, and the PEGylated oligonucleotide was purified by preparative gel electrophoresis. Analyses on gel electrophoresis (Scheme 1) and HPLC confirmed product purities.

**Scheme 1:**
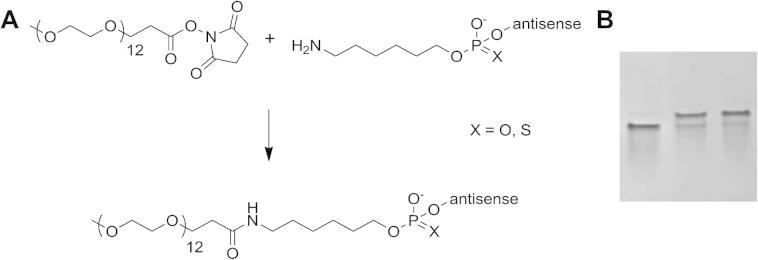
Chemical conjugation scheme of short PEG chains to antisense oligonucleotides. (A) During oligonucleotide synthesis, aminohexyl linkers were attached to antisense oligonucleotides at the 3′- or 5′-terminus. After cleavage and deprotection, the amines were reacted with PEG12-NHS reagent in an aqueous buffer solution. Using a surplus of the PEG ligand, the reaction proceeded nearly quantitatively after 2 h. (B) The attachment reaction was monitored by taking samples with subsequent analyses on a denaturing polyacrylamide gel. The gel shows a representative image of the reaction of a 3′-aminohexyl phosphorothioate antisense oligonucleotide to give conjugate **5** (from left to right; 0 min, 30 min, 120 min reaction time).

Circular dichroism spectra were recorded to detect a possible influence of the ligands on the structural properties of the paired oligonucleotide duplexes. Neither 3′- nor 5′-attachment changed the curve shape (Fig. 1). The slightly higher band intensities even indicate a small stabilization of the duplex structure caused by the PEGylation. Similar results were found for the phosphorothioate oligonucleotides **4** and **5** (data not shown).

**Figure 1:**
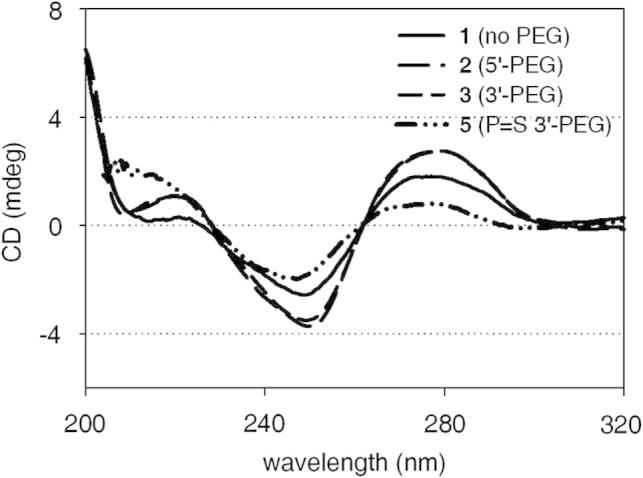
CD spectra of PEGylated antisense oligonucleotide duplexes. The indicated oligonucleotides were mixed in an equimolar ratio with their counterstrand **6** in a tris buffer solution and subjected to circular dichroism analysis.

For an assessment of the target affinity, the duplex transition temperatures (melting temperatures) of the PEGylated oligonucleotides were compared to those of the unmodified ones (Table 2). Generally, only a very minor destabilization was detected for oligonucleotides with phosphodiester backbones (**2** and **3**) compared to non-PEGylated duplexes. For phosphorothioate backbone modified compounds, the transition temperature was about 1 °C higher when the PEG chain was attached. In line with known properties, the melting temperature of phosphorothioates were about 15 °C lower than for their phosphodiester counterparts.^16^ Together with the CD data, it can be concluded that the 3′-and 5′-short PEG ligands do not interfere with recognition and they do not influence the target affinity. Based on the data and the lack of intrastrand modifications, it can be safely assumed that the RNase H activation ability is not affected by the ligands.

**Table 2:** Duplex transition temperatures of PEGylated oligonucleotides.

Oligonucleotide	Melting temperature^a^
**1** **+** **6**	80.73 ± 0.30
**2** **+** **6**	78.30 ± 0.22
**3** **+** **6**	79.40 ± 0.57
**4** + **6**	64.67 ± 0.76
**5** **+** **6**	65.83 ± 0.85
**7** **+** **10**	85.70 ± 0.71
**9** **+** **10**	86.75 ± 1.26

a Values are reported as average ± SEM of at least three independent measurements.

The gene silencing activity was evaluated in vitro with a luciferase reporter assay. In analogy to the reported psiCHECK2-bcl-2 plasmid,^12^ we generated a luciferase reporter plasmid for assaying the silencing effect on galectin-1, a promising oncology target.^17^ It was generated by cloning the human galectin-1 cDNA into the empty psiCHECK2 vector.^12^ The renilla luciferase intensity corresponds to the expression of the target gene, while firefly luciferase is used for normalization of transfection efficiency. For reverse co-transfections of antisense or siRNA oligonucleotides with the psiCHECK2 plasmid, lipofectamine 2000 was used, and luminescence intensities were measured with the Dual Luciferase Assay Kit from Promega. For application of oligonucleotides in the absence of a transfection reagent, the plasmid was reverse transfected, the transfection mix removed after 24 h, and fresh medium containing the indicated amounts of oligonucleotides was added.

Breast cancer cells MCF-7 were treated with both phosphodiesters (**1** and **3**) and phosphorothioates (**4** and **5**) with and without 3′-PEG ligand (Fig. 2). After co-transfection with lipofectamine 2000, all tested oligonucleotides resulted in significant gene silencing (Fig. 2A, *p* < 0.05). Neither statistically significant enhancement nor decrease of the effect was detected by the PEGylation of the phosphodiester backbone compound. In contrast, the PEGylated phosphorothioate **5** showed higher gene silencing activity than its non-PEGylated counterpart **4** after transfection in a concentration of 100 nM (*p* < 0.05).

**Figure 2:**
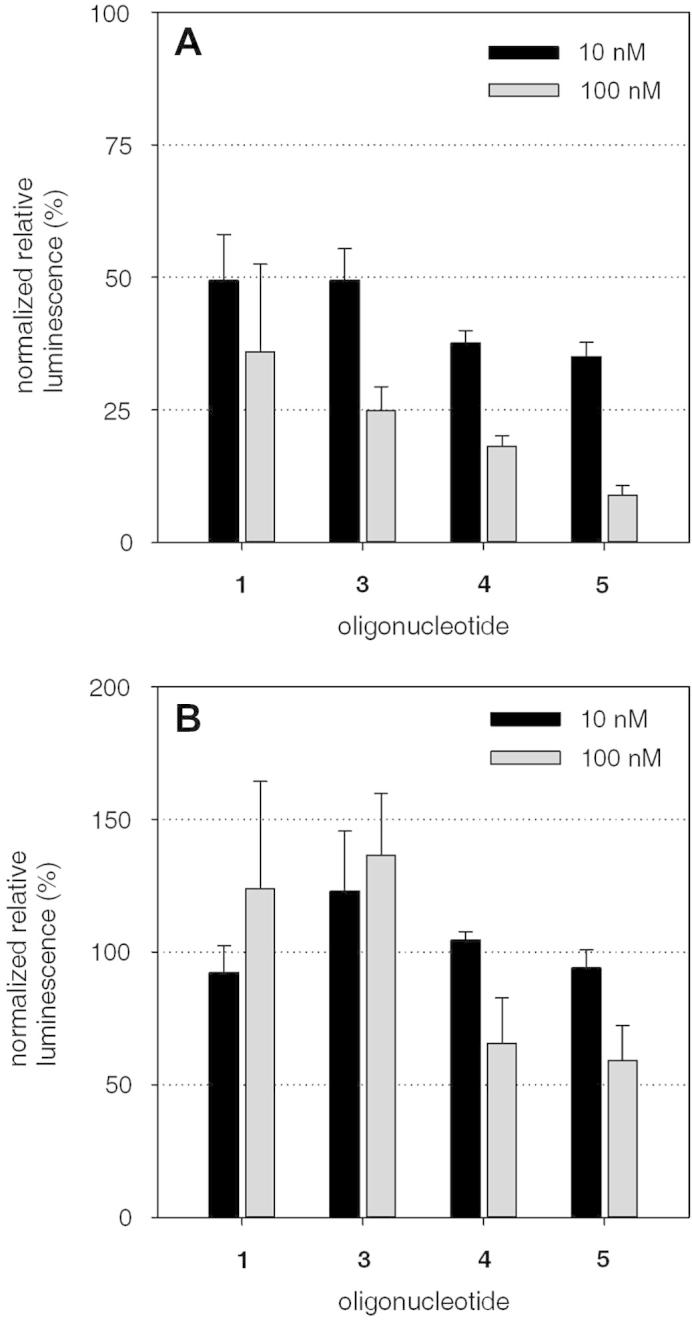
Effect of PEGylated phosphorothioate antisense oligonucleotides in a luciferase reporter assay after transfection with lipofectamine 2000 (**A**) and after naked application (**B**). For transfections, the indicated oligonucleotides were mixed with the psiCHECK-2-Gal-1 plasmid encoding renilla luciferase fused to the human cDNA of galectin-1, and the firefly luciferase for normalization. The mixture was co-transfected into the breast cancer cell line MCF-7 and after 48 h incubation, the luminescence levels were recorded in a plate reader. For the evaluation of unassisted cellular uptake, the plasmid was first transfected into the cells, and 24 h later the oligonucleotides were added into fresh medium. Luminescence levels were analyzed after a 48 h incubation period. Values are reported as renilla normalized to firefly luciferase signals (*n* = 3, error bars are SEM). In co-transfections, the phosphorothioate conjugate **5** showed higher efficacy compared to the isosequential oligonucleotide **4** (100 nM, *p* < 0.05), but no influence of the PEGylation on gene silencing activity was found after naked application (gymnotic uptake).

In order to assess the effect on cellular uptake, the gene silencing efficiency was evaluated after application of the uncomplexed agents (Fig. 2B) in the presence of serum. As expected, all phosphodiesters failed to induce any effect on the expression of the targeted gene, presumably caused by rapid degradation through serum nucleases and the inability of the compounds to permeate cellular membranes. The phosphorothioate oligonucleotides with and without a PEG ligand resulted in moderate target down regulation after treatment in a concentration of 100 nM. In line with their known properties, the phosphorothioate backbone enhances cellular uptake.^14^ Indicated by the same extent of gene silencing by the oligonucleotides **4** and **5**, the polyethylene glycol ligand failed to significantly further enhance the membrane permeation properties of the antisense agent.

To exclude any unspecific or toxic effects, cell proliferation rates were assessed after treatment with the modified oligonucleotides, applied without transfection enhancer (Fig. 3). In concentrations that resulted in successful gene silencing, none of the oligonucleotides reduced proliferation by more than 15%, with a 13% reduction caused by non-PEGylated phosphorothioate **7** (100 nM) being the most pronounced effect. Thus, PEGylation did not induce any negative effect on proliferation, indicating a good in vitro safety profile of the conjugates.

**Figure 3:**
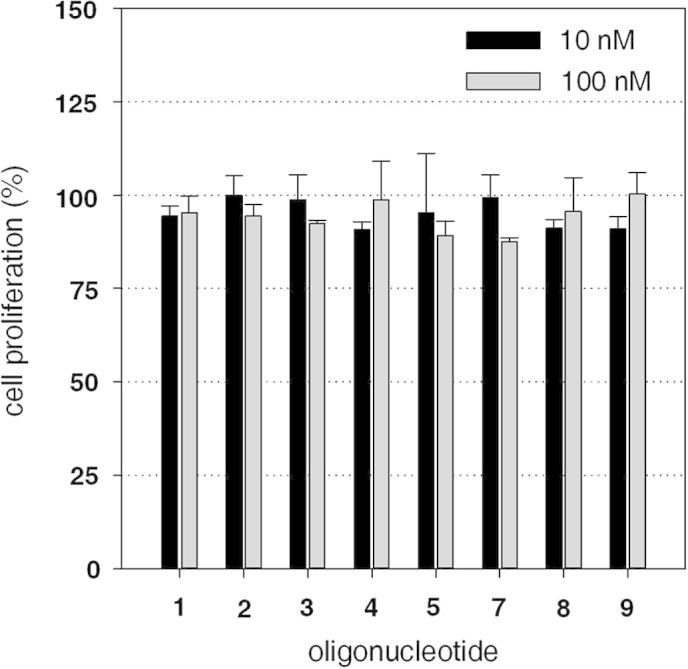
Effect of PEGylated phosphorothioate antisense oligonucleotides on cell proliferation. To evaluate unspecific toxicity, cell proliferation rates were assayed after treatment with antisense oligonucleotides. The indicated compounds were applied without transfection reagents, and proliferation was evaluated after a 24 h incubation period with an MTS assay.^18^ No significant influence on proliferation was detected for any compound, indicating no apparent toxic effects caused by the PEG conjugations (*n* = 3, error bars are SEM).

In total, we have shown that antisense oligonucleotides can be modified with short PEG_12_ chains at the 3′-and 5′-end without any loss in gene silencing activity, indicating a tolerance of the molecular effector RNase H towards this derivatization. Although the unassisted cellular uptake is not significantly enhanced through this short amphiphilic chain, gene silencing was increased after administration in 100 nM concentration when used in conjunction with a transfection agent. This corroborates the results found for siRNA, for which the short PEG ligands improved the effect in some cases.^12^ It can be speculated that these outcomes are caused by higher stability and/or better endosomal escape through the amphiphilic ethylene glycol. The PEG attachment is structurally and synthetically compatible with other chemical modifications, including backbone derivatization, 2′-functionalization and even 3′-or 5′-bioconjugation techniques. This easily introduced modification by itself is not sufficient for significantly improving the pharmacokinetic and pharmacodynamic characteristics of antisense agents, because the short PEG chain will not prevent renal filtration.^11^ Thus, although not suited as a stand-alone modification for delivery, it may be a useful component towards enhancing oligonucleotides by chemical derivatization or in combination with oligonucleotide delivery systems.
